# Supplementary material for: Equity in awareness and utilization of cervical cancer screening services among women of reproductive age in Uganda: Analysis of vertical equity using evidence from UDHS 2022
Source: PLoS One. 2026 Apr 7;21(4):e0335336. doi: 10.1371/journal.pone.0335336 (PMC13056166; doi:10.1371/journal.pone.0335336)
Supplement: Attachment — Submitted filename: Response to Reviewers.docx [file pone.0335336.s002.docx]

**POINT BY POINT RESPONSE TO REVIEWER’S COMMENTS**

| **Reviewer Comment** | **Author Response** | **Changes Made in Manuscript** | **Location in Manuscript** |
| --- | --- | --- | --- |
| **Reviewer 1** | | | |
| Title: Inclusion of Urban and Rural is kind of skeptical because, it's very much known that Uganda has both. I would suggest omission of the words and consider retaining Uganda alone | We fully acknowledge this and have revised the title to exclude Urban and Rural | “Equity in Awareness and Utilization of Cervical Cancer Screening Services Among Women of Reproductive Age in Uganda: Analysis of Vertical Equity Using Evidence from UDHS 2022” | Title |
| **Abstract** Background: While articulating the background information withe objectives, the objective should be written in past tense  Methods: It's important to state at what level was the p-value set to report significance  Results: Here the sentence "National screening utilization was 13%" sounds ectopic because this wasn't part of the objectives and hence shouldn't be reported and as the matter of fact it's reported in the UDHS report  Conclusion: You have reported that "There are stark socioeconomic and geographic inequities in cervical cancer screening in Uganda" Was this a primary goal of your research? I suggest conclusion of the key findings mirroring the study research question | We acknowledge this and have revised the statement as advised | “This study assessed vertical equity in the awareness and utilization……….” | Line 23-24 |
|  | This is agreeable and we have explicitly stated the P.value | “P.value of 0.05 (95% confidence interval) was used…….” | Line 30 |
|  | Thank you for this observation, we have deleted this from our abstract |  | Line 32-36 |
|  | Thank you for your guidance on this, we have re-written our conclusion to stick to the research question | “Uganda's cervical cancer screening services demonstrate significant vertical inequity,” | Line 37-40 |
| **Background:** While it's very well written, I suggest the inclusion of the consequences of not screening for cervical cancer | Thank you for this observation, however this was stated in the manuscript on line 66 | “The direct consequence of this low and uneven screening uptake is catastrophic……..” | Line 66 |
| **Methodology:** Study design: It important to make sure that it's explicitly study design as it's observed that, there more unnecessary information included. The definition of Equity and inequity shouldn't be one this sub-section and wherever appropriately placed it's source should be cited | Thank you for noting this mix-up in the study design, we have now explicitly stated the study design separate from the section on definition of equity with relevant citations | **Study design**: This study employed a cross-sectional design guided explicitly….  **Veridical Equity Framework**: Equity in health refers………. | Line 100  Line 105 |
| **Description of data set**: This should be rephrased to Data source where you will also need to include sampling procedures undertaken | Agreed, we have revised the statement to Data Source |  | Line 126 |
| **Exploration of dataset**: This subsection should be in the analysis plan | This is Agreed, we have included this in the statistical plan |  | Line 184 |
| **Exclusion criteri**a is not the opposite of the inclusion criteria please revise | We fully acknowledge this and have re-written the exclusion criteria | “The study excluded women for whom data on the primary outcomes—cervical cancer screening utilization or awareness—were missing or incomplete, as these records could not contribute to the core analysis.” | Line 141-144 |
| **Independent variables**: Make sure that you have operationalize the variables by including the how they were coded in STATA and why so ? | We fully acknowledge this omission, we have now include how these variables were coded in STAT and why |  | Line 146-259 |
| **Dependent variables**: The word utilization and use are very much different please revisit oxford dictionary and choose the appropriate word. | Thank you for highlighting this important distinction. In public health and health services research, the terms "utilization" and "use" are indeed employed with specific meanings, and precision is crucial.  In this context, "utilization" is the appropriate and standard term. It refers specifically to the actual consumption or uptake of a health service—in this case, the act of undergoing a cervical cancer screening test. This aligns perfectly with our operational definition, which is based on the survey question, "Have you ever been screened for cervical cancer?" and coded as a binary indicator of action taken.  The term "use" is broader and can sometimes imply general employment of a service or method without the specific connotation of measured consumption within a healthcare system. Therefore, retaining "utilization" accurately reflects the study's focus on quantifying real-world access and engagement with the screening service.  We have ensured that the term "utilization" is applied consistently throughout the manuscript (e.g., in the objectives, variable definitions, results, and discussion) to maintain methodological clarity. We appreciate your vigilance in ensuring terminological precision |  | Line 161-169 |
| **Statistical Analysis**: The sentence "Associations between categorical variables were assessed using chi-square tests" is not right. Refer to the book titled "Essential Medical Statistics" written by Betty Kirkwood where its clearly stated that chi-square can not assess the association but rather the distribution of independent variable to the dependent variable | Thank you for this precise and valuable correction. You are absolutely right. The phrasing was statistically imprecise.  Referencing Kirkwood & Sterne's Essential Medical Statistics, the chi-square test is correctly used to determine if there is a statistically significant difference in the distribution of one categorical variable across the categories of another, or to test the independence between two categorical variables. It does not measure the strength or direction of an association in the manner of correlation coefficients | The sentence has been revised for accuracy as follows:  Revised Sentence: "The chi-square test was used to assess for statistically significant differences in the proportions of screening utilization and awareness across the categories of independent variables, such as residence and wealth quintile."  This revised description accurately reflects that the test compares the distribution of the dependent variable (e.g., screening 'Yes'/'No') across the groups of the independent variable (e.g., rural/urban), which was the intended analysis. Thank you for ensuring the methodological reporting is exact. | Line 196-203 |
| **RESULTS:** Well written however: As the rule of thumb do you think its not important to describe your study population as objective zero? | You are absolutely write, this has now been factored in the results section |  | Results |
| **DISCUSSION**: Its also important to discuss the implication of the results as well as comparison from other findings | Thank you for this important suggestion to strengthen the discussion. We have revised the section to more explicitly outline the implications of our key findings and to integrate a clearer comparison with existing evidence. | Specifically, we have added a dedicated paragraph discussing the direct programmatic and policy implications of the observed inequities, arguing that a standard approach is insufficient and that interventions must be dual-pronged. Furthermore, we have expanded the comparison with other studies, more clearly situating our findings on pro-rich and geographic inequities within the broader literature on cervical cancer screening in sub-Saharan Africa. These changes ensure the discussion not only reports our results but also interprets their significance for policy and practice. | Discussion |
| **Reviewer 2** | | | |
| Language & Presentation: Numerous typographical, grammatical, and stylistic errors reduce clarity and readability. Internal inconsistencies in numbers and percentages exist between text and tables. | We fully acknowledge this issue and have undertaken a comprehensive, professional language edit. All figures have been checked for consistency. | The entire manuscript was professionally edited for grammar, spelling, syntax, and flow. All numerical values, percentages, and denominators (e.g., national screening rate, urban/rural breakdowns) were harmonized across the abstract, text, tables, and figures. Terminology was standardized (e.g., consistent use of "utilization," "pro-rich/pro-poor"). | Throughout the entire manuscript. |
| Data Availability Statement: Should be strengthened to fully comply with PLOS ONE policy regarding third-party data. | Agreed. We have revised the statement to be explicit about data ownership, accessibility, and our provision of analysis code. | A new Data Availability section was added, stating the data are third-party DHS data, available upon request from The DHS Program/UBOS, and that analysis code will be shared upon request. An explicit Ethics statement regarding secondary data use was also added. | End of manuscript (before References). Line 356 |
| Methodology - Survey Weights: Must explicitly state whether sampling weights, clustering, and stratification were applied. | Thank you. This is a crucial point for inference. All analyses accounted for the complex survey design. | The Statistical Analysis section was revised to explicitly state that all analyses used the svy suite of commands in STATA, applying provided sampling weights and accounting for stratification and clustering to produce nationally representative estimates and correct standard errors. | Methods: Statistical Analysis |
| Methodology - Concentration Index: Should specify if a corrected index (e.g., Erreygers or Wagstaff) was used for binary outcomes and justify the choice. Confidence intervals for key estimates should be added. | We thank the reviewer for this technical clarification. We used the Erreygers correction and have added CIs. | The Statistical Analysis section now specifies the use of the Erreygers corrected concentration index (ECI) for bounded binary outcomes, with a brief justification. The reporting of 95% confidence intervals for prevalence estimates and concentration indices has been added throughout the Results section (text and tables). | Methods: Statistical Analysis; Results |
| Methodology - Statistical Language: The sentence on chi-square tests imprecisely suggests it assesses "association." | Correct. We have amended the language to accurately reflect that chi-square tests assess differences in distributions or independence. | The sentence was revised to: "The chi-square test was used to assess for statistically significant differences in the proportions of screening utilization and awareness across the categories of independent variables..." | Methods: Statistical Analysis |
| Results & Interpretation: The conclusion should mirror the primary research goals. The description of the study population is essential. | We agree. The conclusion has been reframed, and the study population is described in detail as the foundational "Objective 0." | The Abstract Conclusion was revised to directly reflect the three study objectives within the vertical equity framework. The Results section begins with a Summary of Variables (Table 1) and narrative describing the cohort's composition by wealth, residence, and outcomes. | Abstract; Results: Summary of Variables |
| Discussion & Implications: Should more explicitly discuss the implications of results and compare them with other findings. Avoid causal language. Link specific findings to policy recommendations more clearly. | Thank you. We have strengthened the discussion by integrating comparisons, focusing on implications, removing causal language, and creating a direct thread from findings to policy. | The Discussion was enhanced to: 1) More explicitly compare findings with prior literature on equity in SSA. 2) State the direct implication that "business-as-usual" is insufficient. 3) Replace causal phrasing (e.g., "results in") with associative language (e.g., "is associated with," "contributes to"). 4) The Conclusion and Policy Implications section now explicitly maps key findings (awareness CI > utilization CI; urban-rural disparity) to specific, actionable policy recommendations. | Discussion; Conclusion and Policy Implications |
| Variable Definitions: The "Exclusion Criteria" was not the logical opposite of the "Inclusion Criteria." The terms "utilization" and "use" should be clarified. The operationalization of variables (coding, categorization rationale) should be explained. | We have corrected the exclusion criteria, affirmed the precision of "utilization," and detailed the operationalization of variables. | 1. Exclusion Criteria revised to focus on missing outcome data. 2. A clarification on the use of "utilization" versus "use" was added to the response letter; the term "utilization" is used consistently in the manuscript. 3. The Independent variable subsection now explicitly states the use of the pre-existing v190 wealth quintile variable, explains the PCA-based categorization, and provides the STATA coding (1=Poorest...5=Richest). | Methods: Study Variables |
| Background & Introduction: Suggestion to include the consequences of not screening. The data source description should be rephrased to "Data Source" and include sampling procedures. | Both suggestions have been implemented to improve the background rationale and methodological transparency. | 1. A paragraph detailing the clinical consequences of late-stage diagnosis due to lack of screening was added to the Background. 2. The Methods subsection was retitled "Data Source" and now details the two-stage stratified sampling procedure of the UDHS. | Background; Methods: Data Source |

**Declaration**: All reviewer comments have been addressed thoroughly. We believe the revisions have significantly improved the manuscript's clarity, methodological rigor, and scholarly impact
